# Properties of the SR Ca-ATPase in an Open Microsomal Membrane Preparation

**DOI:** 10.2174/1874091X00802010091

**Published:** 2008-06-09

**Authors:** A Fibich, C Jüngst, H.-J Apell

**Affiliations:** Department of Biology, University of Konstanz, Konstanz Germany

**Keywords:** Membrane fragments, ion binding, fluorescence, electrochromic dyes, pH effects

## Abstract

SR vesicles isolated from rabbit muscle were treated by a SDS incubation and subsequent dialysis to obtain open membrane fragments that allow a direct access to the luminal membrane surface and especially to the ion-binding sites in the P-E_2_ conformation of the Ca-ATPase. The open membrane fragments showed about 80% of the enzyme activity in the untreated membranes. Pump function was investigated by using electrochromic styryl dyes. The kinetic properties of cytoplasmic ion binding showed no significant differences between the Ca-ATPases in SR vesicles and in membrane fragments. From pH-dependent Ca^2+^ binding it could be deduced that due to the SDS treatment the density of negatively charged lipid was increased by one elementary charge per 12 lipid molecules. Major differences between Ca-ATPase from SR vesicles and membrane fragments were the respective fluorescence amplitudes. This effect is, however, produced by dye-lipid interaction and not by pump function. It was demonstrated that time-resolved kinetics may be study by the use of caged compounds such as caged ATP or caged calcium also in the case of the membrane fragments.

## INTRODUCTION

The Ca-ATPase of the sarcoplasmic reticulum (SR) is present in high concentrations in the SR membrane in muscle cells to promote speedy muscle relaxation by pumping Ca^2+^ ions back into the SR until a cytoplasmic concentration of about 100 nM is maintained, which represents the resting state of the muscle fibres. The stoichiometry of the SR Ca-ATPase was determined to be 2 Ca^2+^/2 H^+^/1 ATP [1-3]. The counter transport of H^+^ was under debate for quite a while since the SR has a high permeability for cations other than Ca^2+^. Therefore, electrophysiological experiments were unsuitable to measure charge transport by the ion pump, and H^+^ counter transport was not obvious since no pH gradient could build up due to the high leak conductance. Eventually, reconstitution of the calcium pump in tight lipid vesicles allowed experiments that provided the answer to this open question and established the mentioned transport stoichiometry [2]. In addition, recent considerations based on highly resolved structural details of several conformations of the SR Ca-ATPase provided arguments on the role of protons in the binding sites. It was concluded that the stability of the ion-binding sites in the E2 conformation of the protein is maintained by protons bound to these sites in the absence of Ca^2+^ ions, by neutralizing negatively charged side chains, or by forming hydrogen bonds between side chains [4]. In the E_1_ conformation it was shown that the binding sites are also occupied either by protons or Ca^2+^ ions at physiological pH [5]. These observations provide a genuine reason for the transport of H^+^ ions in the second half of the pump cycle after the Ca^2+^ ions were released from their sites to the luminal aqueous compartment of the SR.

Both structural and functional details are necessary to understand the molecular mechanism of ion transport through the pump. In the case of structural aspects of the SR Ca-ATPase numerous details became available on atomic level during recent years [6-2]. Complementary functional aspect have to be gained by kinetical investigations, such as phosphorylation/dephosphorylation steps or ion binding and release reactions, and their corresponding rate and equilibrium constants. Significant information on the transport mechanism can be determined by time-resolved investigations of partial reactions in the pump cycle of the Ca-ATPase. So far they could be measured, however, only in the E_1_ conformation of the protein in which ion-binding sites are facing the cytoplasm, and thus are directly accessible on the outside of the SR vesicles. In the P-E_2_ conformation concentration-jump experiments of substrate ions are difficult to accomplish with a sufficiently high time resolution since the kinetics may be affected by diffusion of the ions through the membrane to reach the luminal compartment, even if this process is facilitated by a fast-acting ionophore. Therefore, it was desirable to have a membrane preparation available corresponding to the purified microsomal preparations of the Na,K-ATPase [13, 14]. Such an open membrane preparation would be beneficial since unrestricted access to the ion-binding sites in the P-E_2_ conformation would allow time-resolved relaxation experiments with Ca^2+^-concentration and pH jumps as applied before to the cytoplasmic side [5, 15].

Although membrane fragments with diameters in the order of 0.1 to 2 µm diameter cannot be investigated with electrophysiological methods, Ca^2+^ and H^+^ movements into and out of the binding sites of the SR Ca-ATPase can be detected even in open membrane fragments by a fluorescence technique using electrochromic styryl dyes as introduced and applied successfully during the last decade [16, 17]. With reference fluorescence levels of defined states in the pump cycle even the amount of charges in the binding sites can be determined [5]. In this presentation we introduce a method to produce open membrane fragments from SR vesicles, show that the properties of the SR Ca-ATPase in this preparation are essentially the same when compared to the untreated vesicles and characterize the binding properties of the ion sites in both principal conformations of ion pump.

## MATERIALS AND METHODS

The chelator BAPTA (1,2bis(2-aminophenoxy)-ethane-N,N,N9,-tetrasodium salt, B1214), DMNP-EDTA, caged calcium (1-(4,5-dimethoxy-2-nitrophenyl)-1,2- diaminoethane-*N*,*N*,*N*',*N*'-tetraacetic acid, “DM-nitrophen”), and NPE-caged ATP (P^3^-(-(2-nitrophenyl)ethyl)ester-ATP, disodium salt) were obtained from MoBiTec, Göttingen, Germany. Sodium dodecylsulfate (SDS) was obtained from Pierce Chemical Company (Rockford,Ill.), the calcium ionophore A23187 from Boehringer (Mannheim, Germany). Thapsigargin was purchased from Alomone Labs Ltd. (Jerusalem, Israel). The styryl dyes 2HITC and 2BITC [17] were a gift from Dr. H.-D. Martin, University of Düsseldorf, Düsseldorf, Germany. Caged proton (2-methoxy-5-nitrophenyl sulfate sodium, “MNPS.Na”) was synthesized by Karl Janko in our laboratory. All other reagents were of the highest grade commercially available.

Ca-ATPase was prepared in form of SR vesicles from psoas muscles of rabbits by a slightly modified method of Heilmann and collaborators [18]. The whole procedure was performed at temperatures below 4°C. The determination of the protein content of the membrane preparation was performed according to Markwell *et al*. [19]. The most active fractions of the final density gradient separation had a protein content of 2–3 mg per ml. The enzyme activity was determined by the coupled pyruvate kinase/lactate dehydrogenase assay [20]. Background enzyme activity of the isolated preparation was measured by addition of 10 µM thapsigargin that blocked the SR Ca-ATPase completely. The Ca-ATPase-specific activity of the used preparation was 2.92 µmol P_i_ per mg protein and min at 37 °C in the absence and 5.83 µmol P_i_ per mg protein and min in the presence of A23187 which short-circuits the vesicles membranes for Ca^2+^.

Electron-microscopic images were performed in the EM service unit of the Biological Department (University of Konstanz) to visualize the isolated SR vesicles and the open membrane fragments. A transmission EM (Zeiss TEM 912Ω) was used to study negative-stain treated samples on copper grids. The preparations were stained by a 1% solution of ammonium heptamolybdate. Images were taken at a 100.000 fold magnification and analyzed.

Gel electrophoresis of proteins in SR vesicles and membrane preparations was performed according to standard procedures with SDS polyacryl gel electrophoresis. The SDS-denatured protein mixtures were run through 10% polyacryl amide gels and the molar masses were obtained by comparison with marker proteins.

The fluorescence measurements in equilibrium-titration experiments were performed with a self-constructed setup using a HeNe laser with a wavelength of 543 nm (Laser 2000, Wessling, Germany) to excite the fluorescence of the electrochromic dye 2BITC or 2HITC [21]. The emitted light was collected perpendicularly to the incident light, filtered by a narrow-band interference filter (λ_max_ = 589 nm, half width 10.6 nm) and detected by a head-on photo multiplier (R2066, Hamamatsu, Japan). The photo current was amplified by a Keithley current amplifier 427 (Keithley Instruments, Cleveland, OH) and collected by a data-acquisition board of a PC (PCI-9112, Imtec, Backnang, Germany) with sampling frequencies between 1 and 10 Hz. The experimental data were displayed on the monitor, stored and analyzed on the PC. The temperature in the cuvette (2 ml) was maintained by a Peltier thermostat at 20 °C. Due to the fact that in various experiments the Ca^2+^ chelator BAPTA was used the actual free Ca^2+^ concentrations was determined by the program WinMaxC (http://www.stanford.edu/~patton/).

Time-resolved fluorescence responses on pH jumps induced by proton release from caged proton, MNPS.Na, were performed at the same wavelength settings in an appropriate setup as described recently [5]. The same method applies also to Ca^2+^-concentration jump experiments with caged calcium [22], and ATP concentration jump experiments with NPE-caged ATP [15]. The electrochromic fluorescent dye, 2BITC was excited by a 543 nm HeNe laser from the top of the setup. A quartz lens was adjusted to widen the laser beam and to illuminate the whole solution almost homogeneously. The emitted light was collected by the ellipsoidal mirror and reflected into the second focus of the mirror. An interference light filter (589 ± 10 nm) selected the emitted light of the styryl dye before passing the entrance window of a photo multiplier (PM, R928, Hamamatsu Photonics, Japan). An additional UV cut-off filter reduced the effect of the UV-laser flash used to photo-cleave the caged compound. The output current was amplified by an I/V converter and fed into a 12-bit data-acquisition board of a PC with sampling frequency of 100 kHz. The bottom of the cuvette was in contact with a thermostated copper socket (that also stopped the incident light). The temperature was 20 °C. In order to release a substrate from its caged precursor an UV-light flash (mean duration 14 ns, wavelength 351 nm, max. power 6 MW) was generated by an EMG 100 excimer laser (Lambda Physics, Göttingen, Germany) and directed through a quartz lens into the cuvette, illuminating the whole buffer volume. Data output was collected, stored and analyzed on a computer using DASY-lab software.

## RESULTS

### Membrane Purification

Jørgensen introduced in the late 1960s purified microsomal preparations of the Na,K-ATPase from kidneys of various animal species [23]. This approach was the pattern for the development of the procedure applied to SR vesicles isolated from rabbit psoas muscle. Based on the optimized conditions that were elaborated for the Na,K-ATPase of rabbit kidney, the SR vesicles were treated by an SDS incubation with variable protein/detergent ratios. The composition of the incubation buffer was 50 mM imidazole, 2 mM EDTA, and 3 mM ATP at pH 7.5. The SDS concentration was kept constant at 1.9 mM, the protein concentration was varied between 1.9 mg/ml and 2.3 mg/ml, the incubation time was chosen between 10 and 30 min at 25 °C, stabilized in a thermostated water bath. After incubation the specific enzyme activity was determined in the presence and absence of the Ca ionophore A23187 to test for vesicular structures, and in the presence and absence of thapsigargin, a specific inhibitor of the SR Ca-ATPase, to detect the presence of other ATPases. From series of these experiments the optimal purification conditions were found for a protein concentration of 2.2 mg/ml, and an incubation time of 15 min at 25 °C. A comparison of the specific activity of untreated SR vesicles and the optimized open membrane preparation is shown in Fig. (**1**) for a typical preparation. A23187 could not increase the enzyme activity in the case of the open membranes but produced reproducibly a minor reduction (< 10 %). This fact indicates that no longer vesicular membranes were present, since in the SR vesicles the luminal accumulation of Ca^2+^ reduces the enzyme activity which can be restored by short-circuiting the membrane for Ca^2+^ with A23187. After addition of thapsigargin the residual, unspecific ATPase activity was about 1% of the uninhibited activity. This negligible amount was similar in the SR vesicles and the open membranes.

In the case of the SR vesicles used for the presented experiments the specific enzyme activity was 5.83 ± 0.1 µmol Pi per mg and min in the presence of A23187 and at 37 °C. The open membranes had a specific activity of about 3.67 ± 0.3 µmol Pi per mg and min in the presence of SDS. After the detergent was removed from the incubation solution by dialysis in a dialysis tubing (Visking^®^, dialysis tubing 8/32) for three days against an 1000-fold excess volume containing 8.8 mM sucrose and 20 mM Trizma-maleate, pH 7.0, enzyme activity increased to 5.0 ± 0.2 µmol Pi per mg and min as determined from ten different preparations. (Errors are given in s.e.m.)

### Characterization of the Open Membrane Preparation

The lipid and protein content of the open preparation was estimated by determination of the protein content with the Markwell method [19], and the content of lipids by the enzymatic phospholipid B test [24]. This test provides the contents of phosphatidylcholine lipids (PC). Typical ratios of protein/PC (w/w) were 8.0 (compared to the SR vesicles: 2.6). Assuming a PC content of 68.4% of the total lipid [25], the protein/lipid ratio would be 5.5 in the open membranes. The purity of the Ca-ATPase in the open membranes was checked by SDS gel electrophoresis as shown in Fig. (**2A**). The dominant band of the gels corresponded to the molar mass of the SR Ca-ATPase [26]. No significant differences in sequence and density of protein bands could be detected between proteins from the SR vesicles and the open membrane fragments. This indicates that the SDS treatments primarily removed lipids from the SR membrane, and this process led to membrane preparations in which the protein density became so high that no longer a curvature of the membrane was possible that is needed to form vesicular structures.

Electron-microscopical images in negative stain of the native SR membrane vesicles and the open membrane preparation are shown in Fig. (**2B** and **2C**). The SDS treatment of the more or less spherical vesicles and the subsequent dialysis produced irregularly shaped flat membrane fragments with diameters of 50 to 200 nm with a slightly elevated (unstained) edge, correspondingly to what was observed with membrane fragments from the Na,K-ATPase before [27].

### Kinetical Properties of the Ca-ATPase in the Open Membranes

To test functional integrity of the SR Ca-ATPase experiments with the electrochromic styryl dye 2BITC were performed with both native vesicles and open membrane fragments produced from the same preparation. Since binding (or release) of ions to (or from) the SR Ca-ATPase is electrogenic, these reaction steps can be detected by the fluorescence of styryl dyes [17].

**Fig. (3):**
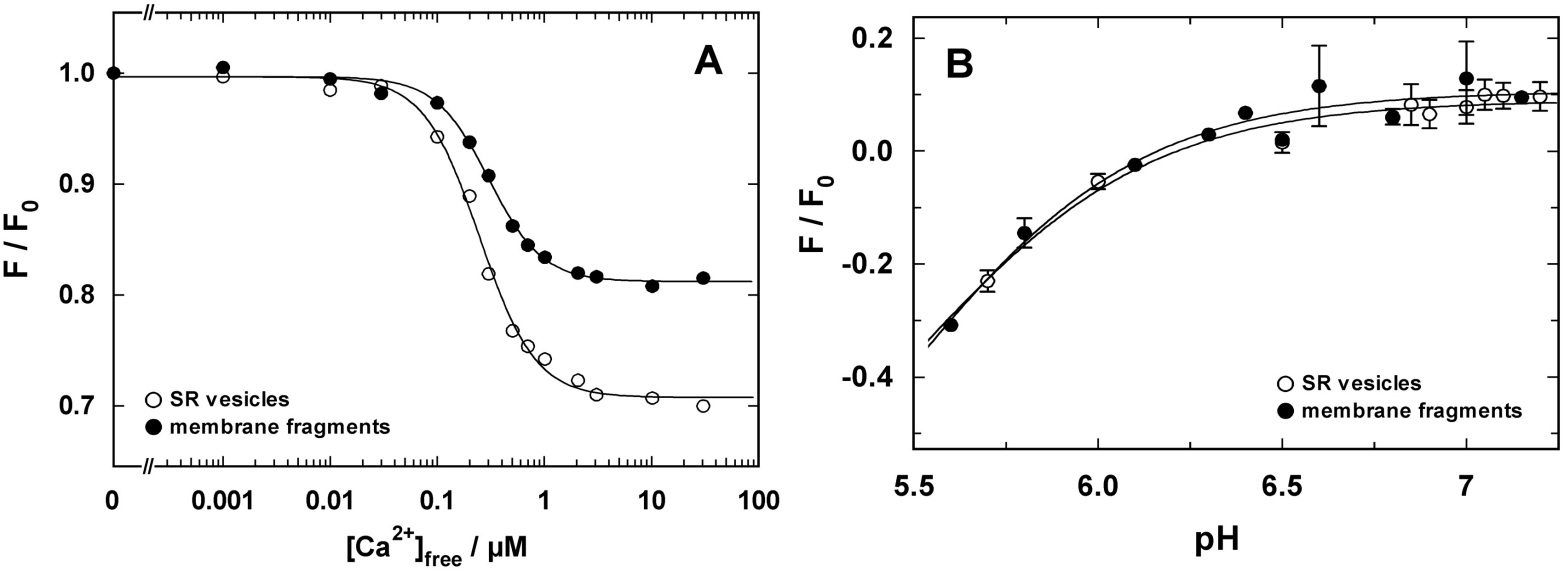
Comparison of Ca^2+^ and H^+^ binding to the SR Ca-ATPase in SR vesicles (open circles) and open membrane arations (solid circles). (**A**) The experiment was performed at pH 7.2. The fluorescence intensities were normalized to the level in the nominal absence of Ca^2+^. The normalized fluorescence amplitude was plotted against the calculated free Ca^2+^ concentration in the buffer solution. The data points were fitted with a Hill function (Eq. 1). The half saturating Ca^2+^ concentration was 0.25 µM (vesicles) and 0.3 µM (membrane fragments). The Hill coefficients were 1.7 in both cases, and the maximum fluorescence changes was 0.3 for the vesicle preparation and 0.19 for the open membrane fragments. (**B**) pH titration in the absence of Ca^2+^ was started in buffer adjusted to pH 7.3-7.4 and performed by addition of small aliquots of HCl. pH was measured after each addition with a pH microelectrode. The lines hrough the data sets are Hill fits that show the close proximity of the H^+^ binding properties of the SR Ca-ATPase in both preparations

In a first set of experiments binding and release of H^+^ and Ca^2+^ were studied by equilibrium titration experiments in the E_1_ conformation of the ion pump. In a cuvette buffer containing 25 mM 3-(N-Morpholino)-propanesulfonic acid (MOPS), 1 mM MgCl_2_, 50 mM KCl, 200 mM choline chloride, pH 7.2, were equilibrated at 20 °C with 200 nM 2-BITC and 18 µg/ml protein in form of SR vesicles or open membranes. The contamination of this aqueous solution with Ca^2+^ was about 4 µM [16]. After a stable fluorescence level was obtained, 200 µM BAPTA were added to deplete the buffer of Ca^2+^ (~1 nM free Ca^2+^), and in consequence, to remove quantitatively the residual Ca^2+^ ions from the binding sites. The removal of Ca^2+^ was observed by an increase of the fluorescence level according to the detection mechanism of 2-BITC [21]. This level was used as reference level, *F*_0_, for the titration experiments. Then appropriate aliquots of CaCl_2_ solutions were added. The fluorescence levels, *F*([Ca^2+^]), were normalized respective to *F*_0_ and plotted against the corresponding free Ca^2+^ concentration (Fig. **3A**). Such experiments were performed with SR vesicles and open membrane fragments. In both cases the concentration dependence was fitted by a Hill function,

$\frac{F\left(\left[{\mathrm{Ca}}^{2+}\right]\right)}{F_{0}}=\;1-\frac{\Delta F/F_{0}}{1+{\left(K_{M}/\left[{\mathrm{Ca}}^{2+}\right]\right)}^{n}}$

While in both experiments the half-saturating Ca^2+^ concentrations, *K*_M_, and the Hill coefficient, *n*, were almost the same (see figure legend), a significant difference was observed for the maximum fluorescence decrease, Δ*F*/*F*_0_, which was 30% for the SR vesicles and about 20% for the open membranes. The relative fluorescence change of 2-BITC depends on the one hand on the amount of charge per area inside the membrane domain of the membrane, i.e. in the binding sites, and on the other hand it depends also on the dielectric coefficient of the membrane, which is controlled by the lipid composition and the protein/lipid ratio. Since the SDS treatments modifies both properties, it is not surprising that the fluorescence change, Δ*F*/*F*_0_, is altered. This is, however, a property of the lipid domain of the membrane and not of the ion pumps, and therefore not significant for an analysis of the pump kinetics.

Corresponding pH titration experiments were performed in the nominal absence of Ca^2+^ in buffer containing 25 mM MOPS, 1 mM MgCl_2_, 50 mM KCl, and a starting pH of 7.3-7.4, adjusted by addition of KOH. After equilibration with 200 nM 2-BITC and 18 µg/ml protein in form of SR vesicles or open membranes pH titrations were performed by addition of small aliquots of HCl. The results are shown in Fig. (**3B**). The experimental data were fitted by a Hill function corresponding to Eq. (1) for the H^+^ concentration. The obtained pK values were 5.5 (SR vesicles) and 5.46 (membrane fragments), although they are not really significant since the data do not cover a large enough pH range to justify the determination. The fits allow us, however, to visualize the close agreement of the H^+^-binding properties of both preparations.

**Fig. (4):**
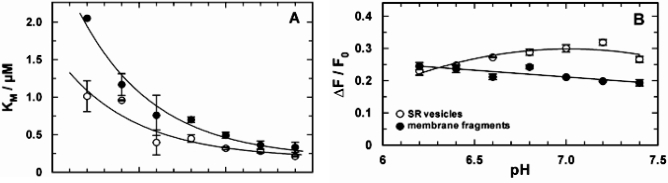
Comparison of the pH-dependent Ca^2+^ binding in the E_1_conformation of the SR Ca-ATPase. Titration experiments as shown in fig. (**3**) were analyzed and the characteristic fitting arameters in the Hill function, *K*_M_ and Δ*F*/*F*_0_,were plotted against the pH of the buffer solution in the cuvette. (**A**)The half-saturating Ca^2+^ concentration,* K*_M_,increased in both preparations with lower pH, indicating a competition between Ca^2+^ and H^+^ in the binding sites. At high pH the *K*_M_ of both preparation merges at 188 nM. The data are fitted with an exponential curve as expected for the pH dependence of the *K*_M_ for Ca^2+^ value when competitive inhibition by protons is occurring (cf.Discussion). The plotted fit curves differ only in a pH shift of 0.29 between both data sets. (**B**) The fluorescence change upon addition of saturating Ca^2+^ (here plotted as absolute value) is larger for the experiments with SR vesicles above pH 6.5.This effect is mainly cause by a difference in the lipid composition of the membranes and does not affect significantly the ion-pump kinetics.

Similar equilibrium titration experiments with CaCl_2_ were performed at various buffer pH between pH 6.4 and 7.8. At each pH at least 3 experiments were performed. In Fig. (**4**) the comparison of the results between Ca-ATPase in SR vesicles and in open membrane fragments are shown. The increase of *K*_M_ with decreased pH (Fig. **4A**) is in agreement with the competition between Ca^2+^ and H^+^ for the same binding sites [5].

If it is assumed that Ca^2+^ binding and competition of H^+^ and Ca^2+^ in the binding sites is unaffected by the SDS treatment, the pH dependence of the apparent K_M_ in both data sets can be fitted by the same exponential function (solid line in Fig. **4A**). The equilibrium dissociation constant at high pH (for both curves) was found to be 188 ± 60 nM. The curve for the data obtained with SR vesicles is shifted by ΔpH of 0.25 to the left. This can be interpreted as the effect of a higher density of negative charges on the cytoplasmic surface of the membrane fragments which causes an apparent higher proton concentration (or lower pH) according to the Gouy-Chapman theory. A quantitative analysis (not shown) shows that a pH shift of 0.29 is generated by a difference of the surface-charge density of one elementary charge per 8 nm^2^. Assuming an average cross-sectional area of a lipid molecule in the lipid bilayer of 0.64 nm^2^, 15 lipid molecules fit in 10 nm^2^, and the membrane fragments, therefore, may be expected to have one additional negatively charge per 12 lipid molecules compared to the isolated SR membrane.

In the case of SR vesicles the maximum fluorescence change (see Fig. **3A**) is significantly larger at buffer pH 7 and above (Fig. **4B**). The Hill coefficient obtained from the fits of the Ca^2+^ titration curves was in the average 2.4 ± 0.4 (SR vesicles) and 1.5 ± 0.1 (membrane fragments).

These results indicate that the kinetical properties of the binding sites in the E_1_ conformation were not significantly altered by the SDS treatment of the SR vesicles to obtain the open membrane fragments.

Similar equilibrium titration experiments were performed also in the P-E_2_ conformation of the Ca-ATPase. These experiments were performed in a buffer containing 25 mM MOPS, 1 mM MgCl_2_, 50 mM KCl, 200 mM choline chloride, 200 nM 2-BITC, 18 µg/ml enzyme prep, 200 µM BAPTA, 12.5 µM A23187, and 200 µM Na_2_ATP. The Ca^2+^ ionophore A23187 was necessary to allow an equilibration of Ca^2+^ across the membrane in the case of SR vesicles. In the presence of Ca^2+^ ions and ATP in the aqueous electrolyte the ion pump works under turnover conditions. It is known, however, from recent studies [21] that the return from the E_2_P to the E_1_ conformation is slow. Therefore, a predominant fraction of the enzyme is present in its phosphorylated conformation and allows titration experiments with the ion-binding sites accessible from the luminal side of the membrane. Experiments were performed in buffer solutions with a pH between pH 6.2 and 7.4. 

**Fig. (5):**
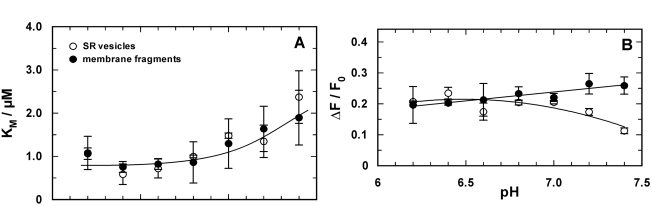
Comparison of the pH-dependent Ca^2+^ binding in the E2P conformation of the SR Ca-ATPase in which the ion-binding sites are accessible from the luminal side of the membrane. Titration experiments were analyzed and the characteristic fitting parameters in the Hill function, K_M_ and &ΔF/F0, were plotted against the pH of the buffer solution in the cuvette. **(A)** The half-saturating Ca^2+^ concentration, K_M_, from both preparation did not significantly differ from each other over the pH range covered by experiments.Below pH 7 it was approximately constant with a value of 0.86±0.06 µM and increased to about 2 µM at pH 7.4. In contrast to the results in the E1 conformation no competition between Ca^2+^ and H^+^ is evident. **(B)** At pH 7 and below the fluorescence change upon addition of saturating Ca^2+^ (here plotted as absolute value) is the same for the experiments with both preparations. The decrease of ΔF/F0 in the case of the SR vesicles may not be significant, it was not found before [21]

The titration curves (not shown) were analyzed using the Hill function, Eq. (1), and the results are shown in Fig. (**5**). The obvious differences from the results obtained in the E_1_ conformation (Fig. **4**) are an additional indication that the binding sites of the ion pump were probed in the E_2_P conformation. The pH dependence of the half-saturating Ca^2+^ concentration, *K*_M_, was the same in both membrane preparations (Fig. **5A**). No significant pH dependence was observed below pH 7, while above pH 7.0 the values of *K*_M_ increased from about 1 µM to 2 µM.

The maximum fluorescence decrease, Δ*F*/*F*_0_, (Fig. **5B**) was 0.22 ± 0.01 for the open membrane fragments throughout (with a slight tendency to increase at higher pH). In the case of the SR vesicles a deviation from the fluorescence changes of the membrane fragments was found only at high pH > 7. The Hill coefficient of 0.84 ± 0.04 was pH independent and the same for both membrane preparations (not shown).

### Time-Resolved Experiments

In a final series of experiments time-resolved concentration-jump experiments were performed by substrate release from caged compounds as published recently [5, 15, 22]. For each substrate, H^+^, Ca^2+^, and ATP, corresponding experiments were performed with SR vesicles and membrane fragments to compare the response to the stepwise increase of concentration upon the flash-induced release from the inactive caged precursor. Typical experiments are shown in Fig. (**6**). The kinetic parameters are shown in Table **1**.

The electrolyte for pH-jump experiments contained 50 mM KCl, 200 mM choline chloride, 400 µM BAPTA, 200 nM 2BITC, 18 µg/ml Ca-ATPase in form of SR vesicles or membrane fragments, and 300 µM MNPS.Na. The initial pH was set to 7.0 by addition of KOH. Five identical experiments were averaged to increase the signal/noise ratio. The time courses of the fluorescence signal for both preparations are shown in Fig. (**6A**). Similar to what was found in equilibrium titration experiments, the amplitude of the fluorescence decrease was smaller in the case of membrane fragments. Both signals could be fitted with the sum of two exponentials

$F(t)=F_{1}\times e^{\left(-t/\tau_{1}\right)}+F_{2}\times e^{\left(-t/\tau_{2}\right)}+F_{\infty }$

The time constants were comparable (*τ*_1_ = 1.4 ms and *τ*_2_ = 31.9 ms for SR vesicles and *τ*_1_ = 2.5 ms and *τ*_2_ = 34.2 ms for membrane fragments). The amplitudes differed significantly. In the case of SR vesicles the contribution of both processes were *F*_1_ = 0.03 and *F*_2_ = 0.052. In contrast the amplitudes were *F*_1_ = 0.025 and *F*_2_ = 0.002 in the case of membrane fragments. It is obvious that the slower process did not significantly contribute to the fluorescence signal in the experiments with membrane fragments.

**Fig. (6):**
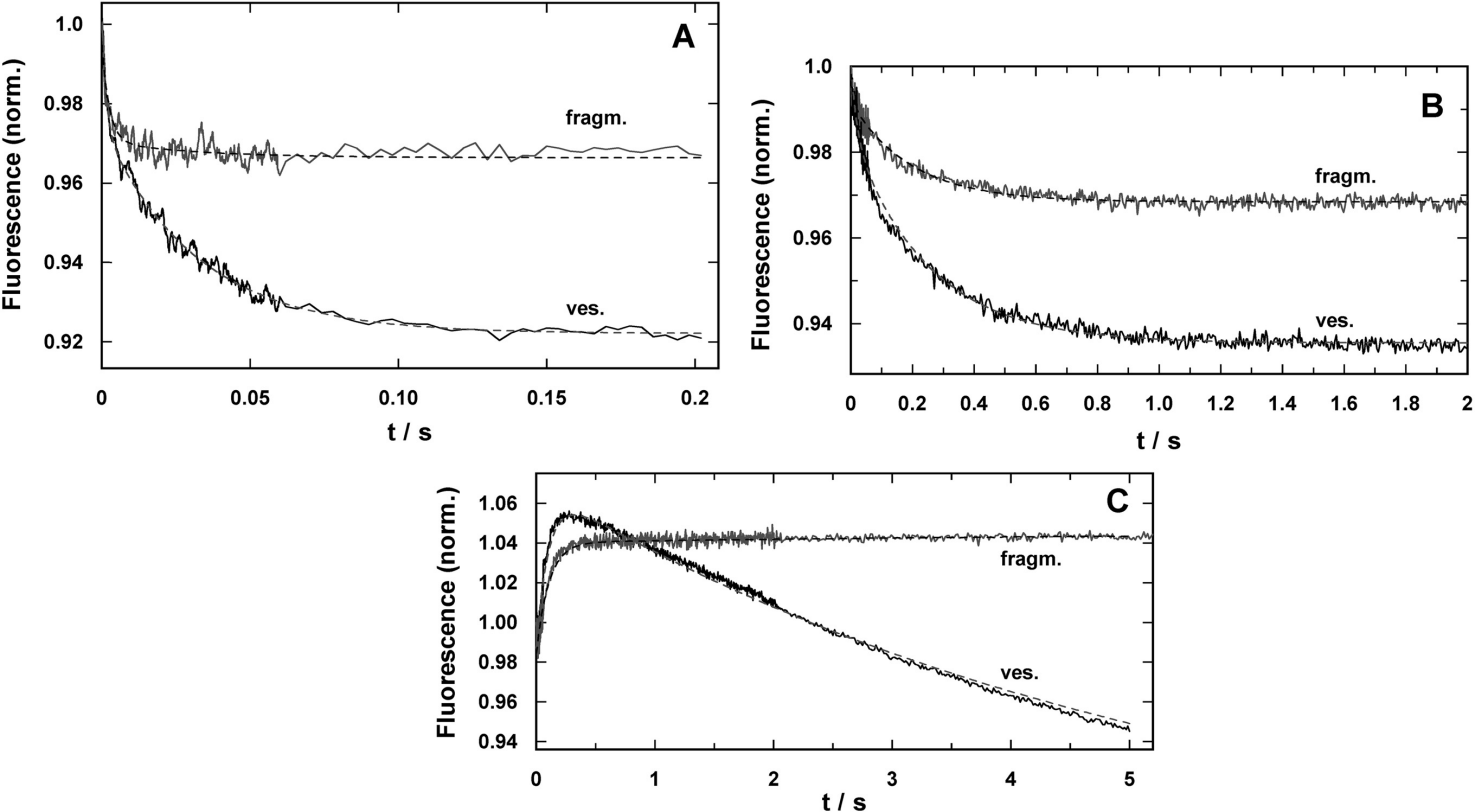
Time-resolved response of the fluorescence signal in concentration-jump experiments performed by UV-flash induced substrate release from a caged precursor with SR vesicles (ves.) and purified membrane fragments (fragm.). **(A)** pH jump ex-periment in the electrolyte that maintains the SR Ca-ATPase in its E_1_ conformation. The release of protons causes a right shift in the reaction sequence, E_1_ ↔ H_2_E_1_ ↔ H_4_E_1_ [5]. In both preparations the time course could be fitted with a sum of two expo-nential functions (Eq. 2). While the time constants were comparable, the amplitudes differed significantly, F_1_/F_2_(ves.) = 0.5 and F_1_/F_2_(fragm.) = 5. **(B)** Ca_2+_-concentration jump in the E_1_ conformation of the SR Ca-ATPase lead to a right shift in the reaction sequence, E_1_ ↔ CaE_1_ ↔ Ca_2_E_1_. Again, the fits of the data with Eq. (2) revealed comparable time constants, the ampli-tude ratios, F_1_/F_2_(ves.) = 0.28 and F_1_/F_2_(fragm.) = 0.21, were not to far from each other, however, the total fluorescence ampli-tude differed by more than a factor of 2. **(C)** ATP-jump experiments were performed under the condition that release of the nucleotide triggered the reaction, Ca_2_E_1_ → (Ca2)E_1_-P → P-E_2_(Ca2) → P-E_2_, and then all substrates are present to allow pump turnover, controlled by the rate-limiting step, P-E_2_ → P-E_2_H_2_ [22]. While the SR vesicles show a biphasic behavior the mem-brane fragments exhibit only the rising phase of the fluorescence with a time constant similar to that for the Ca-ATPase in the vesicles

The response on Ca^2+^ concentration jumps was measured in a buffer containing 50 mM Hepes, 100 mM KCl, 600 nM 2BITC, 25 µM CaCl2, 18 µg/ml Ca-ATPase in form of SR vesicles or membrane fragments, and 25 µM caged calcium approximately saturated with Ca^2+^ (free [Ca^2+^] < 100 nM), pH 7.0. Five identical experiments were averaged to increase the signal/noise ratio (Fig. **6B**). Again, the amplitude of the fluorescence decrease was smaller in the case of membrane fragments. When fitted with Eq. (2) the fluorescence amplitudes were: *F*_1_ = 0.021 and *F*_2_ = 0.041 (SR vesicles), and *F*_1_ = 0.005 and *F*_2_ = 0.02 (membrane fragments). The ratio *F*_1_/*F*_2_ was 0.51 in the case of the SR vesicles and 0.25 in the case of membrane fragments. The relative contribution of the faster of both processes is somewhat reduced in the case of membrane fragments. The time constants of both processes induced by the Ca^2+^ concentration jump were similar (*τ*_1_ = 44.3 ms and *τ*_2_ = 302 ms for SR vesicles and *τ*_1_ 41.9 ms and *τ*_2_ = 272 ms for membrane fragments).

The most significant differences between SR vesicles and membrane fragments were found in time-resolved ATP-jump experiments. The experiments were performed in electrolyte containing 25 mM Tricine, 50 mM KCl, 1 mM MgCl_2_, 400 µM BAPTA, CaCl_2_ to adjust 2 µM free Ca^2+^, 200 nM 2BITC, 18 µg/ml Ca-ATPase, 100 µM caged ATP pH 7.0. Typical experiments are shown in Fig. (**6C**). The traces shown are each the average of three identical experiments. In the case of the SR vesicles a biphasic signal was found with a rising and falling phase as published before [22]. The time constants determined were *τ*_1_ = 102 ms for the raising phase (*F*_1_ =****0.083) and *τ*_2_ = 5.4 s for the falling phase (*F*_1_ =****0.2). In the corresponding experiment with the membrane fragments essentially a single raising process was found with a time constant of *τ*_1_ = 105 ms (*F*_1_ =****0.055), and an additional minor increase of the fluorescence with *τ*_2_ > 6 s (*F*_1_ ≈****0.004). A similar behavior was found with SR vesicles only at buffer pH > 8 [22].

## DISCUSSION

Due to the recent progress in structure analysis of the SR Ca-ATPase [4, 7, 8, 10-12] an appropriate kinetical investigation of the calcium pump is desirable to correlate structure and function to develop a complemented understanding of the molecular mechanism of ion transport. Recent investigations of the kinetics of ion-transport reaction steps were focused on those which are related to substrate interactions from the cytoplasmic side of the membrane [ 5, 15, 16, 21, 22, 28, 29]. The reason for such a restriction is based on the available membrane preparations isolated from muscle cells, which are vesicles from the sarcoplasmic reticulum. These vesicles can be separated easily from homogenized muscle cells. They are a preparation of cytoplasmic-side outward oriented membranes. In consequence, the luminal binding sides (and their access channels) are not directly accessible. Although protons equilibrate rather fast across the leaky membrane and also the Ca^2+^ permeability can be enhanced significantly by application of the Ca^2+^ ionophore A23187, time resolved studies of the kinetics may be rate-limited by processes different from those of the pump function. In addition, due to the small internal volume of vesicles with a diameter of 100-200 nm (*V* = 0.5 – 4.2×10^-18^ l) the buffering capacity of the head groups of the lipid molecules in the luminal leaflet of the membrane may not be neglected inside the vesicles (nominally *c*_lipid_ = 80 – 150 mM). Therefore, an effort was undertaken to produce a membrane preparation in which the luminal side of the membrane is directly accessible. Such a preparation was introduced in the case of the Na,K-ATPase by the purified microsomal preparation.

The biochemical attempt to conduct a treatment analogously to that used for Na,K-ATPase-containing membranes, namely an SDS incubation to solubilize part of the membrane components, and subsequent dialysis against a detergent-free aqueous medium was successful and could be optimized. It resulted in a preparation of open, flat membrane sheets with active Ca-ATPase. The residual enzyme activity of the so-called purified preparation was about 80% of the Ca-ATPase in the untreated SR vesicles. There are several possible reasons for the reduced enzyme activity. A self-evident one is first of all inactivated and denatured ATPase molecules at the edge of the membrane fragments where the pumps have to be in contact with SDS molecules that form the boundary of the hydrophobic core of the fragmented membranes. A second reason is the modified lipid composition of the purified membrane fragments. From the determination of the lipid contents of vesicles and membrane fragments it could be estimated (with a protein mass of 100.000 g/mol and average lipid mass of 760 g/mol) that in the vesicles ~55 PC-lipid molecules per pump were present. This number was reduced to ~16.4 PC molecules per pump in the case of the membrane fragments. It is this reduced lipid contents that decreases the membrane elasticity and bending, and therefore, prevents vesicle formation. Since different lipid species are expected to be removed by SDS with different effectiveness, the lipid composition of the purified membranes may be significantly altered and either reduce their enzyme activity or even block part of the ion pumps. A third reason, a high residual SDS concentration in the membrane fragments can be ruled out since an increase of the duration of dialysis by a factor of two did not enhance but reduced enzyme activity (data not shown).

How far the SR Ca-ATPase in the membrane fragments was affected by the purification method was investigated by comparison of kinetical properties of the ion pumps in SR vesicles and membrane fragments. By equilibrium titration experiments cytoplasmic Ca^2+^ and H^+^ binding was measured and the results compared between both preparations. In the absence of ATP and P_i_ the pumps are confined to the states in reaction sequence, Ca_2_E_1_ ↔ CaE_1_ ↔ E_1_ ↔ H_2_E_1_ ↔ H_4_E_1_ [5]. The distribution between the various states can be modified by buffer pH and Ca^2+^ concentration. The results are shown in Figs. (**3** and **4**). It turned out that the equilibrium dissociation constants for both ion species were not significantly different. When Ca^2+^ titrations were performed at different pH, notable deviations were found at pH < 7.0 (Fig. **4**). As described in the results section, these differences may be described by a higher negative charge density of the (cytoplasmic) membrane surface in the case of the membrane fragments which is not an unexpected condition after an SDS incubation. The only significant difference in the characteristics of both preparations is the fluorescence levels, *F*/*F*_0_, obtained in the Ca^2+^-titration experiments at pH values above 6.5.

As discussed and verified before [17], the fluorescence amplitude of electrochromic dyes is proportional to the local electric field in the hydrophobic interior of the membrane. And the electric field strength is proportional to the number of charges in the binding sites of the ion pumps which are located in the hydrophobic core of the membrane. The absolute fluorescence, *F*, and the fluorescence changes, Δ*F*/*F*_0_, depend, however, due to basic physical principles, not only on the amount of charge in the binding sites but also on the dielectric constant, *ε*, of the membrane. When the protein density is increased, as in the case of the membrane fragments, *ε* will be also increased. This property reduces the spatial range of the electric field and may reduce the fluorescence alteration, Δ*F*/*F*_0_, upon a change in the occupation of the ion-binding sites compared to the untreated SR vesicles. Another mechanism that may affect the fluorescence response, Δ*F*/*F*_0_, is the position of the dye molecules in the membrane [17]. A tighter protein packing may affect also the depth of insertion of the dye molecules into the hydrophobic membrane core, and tiny shifts in the Angstrom range can cause the observed reduction in the fluorescence response. Finally, it has to be mentioned that in the case of the membrane fragments the dye inserts from both sides into the membrane, in contrast to the vesicles, in which 2BITC enters only into the cytoplasmic leaflet of the lipid bilayer, since the inner leaflet is not accessible, and a flip-flop of the polar head of the dye molecule across the membrane is negligibly small.

The time-resolved concentration-jump experiments demonstrated that the kinetics of the cytoplasmic ion binding was not significantly different in both membrane preparations (Table **1**). The differences in the relaxation-time constants did not vary stronger than within a series of experiments performed with the same preparation [5, 15]. Clear differences were found only in the fluorescence amplitudes of corresponding relaxation processes in both preparations. As has been discussed above, these discrepancies have to be assigned to properties of the lipid matrix of the membrane. An intriguing disagreement was found, however, in the case of the ATP-induced partial reaction (Fig. **6C**) which shows after the initial fluorescence rise an explicit decrease in the case of the vesicle preparation which is absent in the experiments with the membrane fragments. The time constants of the rising phase is in both experiments the same. The comparable fluorescence rise in both preparations indicates a release of positive charge from the binding sites which is caused by the last step in the ATP-induced partial reaction, Ca_2_E_1_ → (Ca_2_)E_1_-P → P-E_2_Ca_2_ → P-E_2_. This sequence is obviously unmodified by the formation of the open membrane fragments. By contrast, the subsequent reactions differ. In a previous study [22] the fluorescence decrease in the vesicle preparation was assigned to proton binding, P-E_2_ + *n* H^+^ → P-E_2_H_n_, since the descent could be controlled by buffer pH and disappeared at pH 8 in the extra-vesicular aqueous phase. Due to the high leak conductance of the SR membrane for protons, an equilibrium between inside and outside could be assumed after the incubation time before the experiments were performed. The absence of H^+^ binding observed in the experiments with open membrane fragments might be caused by apparent pH differences on the luminal side of the membrane. However, in Fig. (**5A**) the pH-dependent Ca^2+^ binding behavior from the luminal side in the P-E_2_ conformation is the same in both membrane preparations, and therefore differences in apparent pH at the membrane surface may be excluded. A possible proposal are modifications in the Ca-ATPase that lead to changes in the binding sites that affect proton binding affinities. The investigation of functional and mechanistic details is subject of running and forthcoming experimental studies.

In summary, it can be stated that the differences in the fluorescence amplitude of both membrane preparations in the corresponding experiments shown can be assigned to interactions between the dye 2BITC and the lipid matrix of the membranes, and they do not reflect modified properties of the Ca-ATPase in the purified membrane fragments. Therefore, the introduced methods provides a membrane preparation that will be the basis of forthcoming studies of the kinetic properties of the luminal binding sites and their access channel which are functional active in the P-E_2_ conformation of the SR Ca-ATPase by time-resolved concentration-jump experiments.

## Figures and Tables

**Fig. (1):**
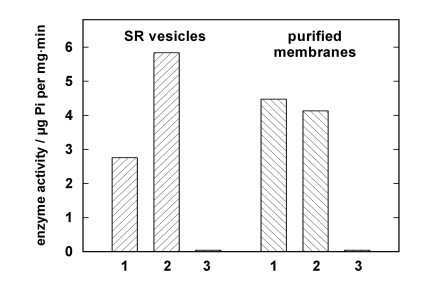
Comparison of enzyme activities in isolated SR vesicles and open membrane patches from a single preparation. Enzyme activity was determined by the coupled pyruvate kinase/lactate dehydrogenase assay [20]. In both columns 1 enzyme activity is shown without further additions, in columns 2 the activity is shown after addition of 12.5 µM of the Ca ionophore A23187. In the case of the SR vesicles a significant increase of the enzyme activity was observed due to the disappearance of the Ca^2+^ concentration gradient across the SR membrane. As expected, in the open mem-branes the ionophore had no amplifying effect. In columns 3 the action of the specific inhibitor thapsigargin is demon-strated. The negligible remaining enzyme activity proves that no significant amounts of other ATPase are present in both preparations.

**Fig. (2):**
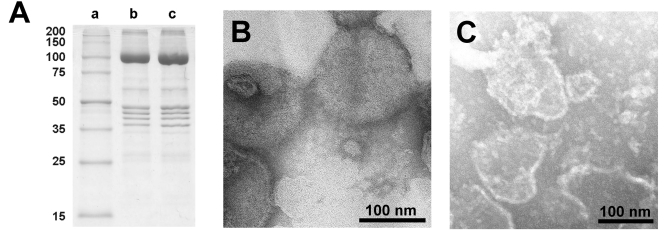
Characterization of the open membrane preparation. (**A**) SDS gel electrophoresis of SR vesicles and the membrane preparation obtained therefrom; lane a: marker enzymes, lane b: untreated SR vesicles, lane c: membrane fragments. The SDS treated membranes exhibit no significant change in the protein composition with respect to the SR vesicles. The prominent protein is the SR Ca-ATPase with a molecular mass of about 100 kDa. (The mass calculated from the 997 amino acids is 109.763 Da.) Electron-microscopic images of (**B**) SR vesicles and (**C**) open membranes obtained with negative stain technique. While the SR vesicles show globular structures with typical diameters of 100 – 200 nm, the open membranes showed irregularly shaped patches with diameter between 50 nm and 200 nm.

**Table 1:** Comparison of the Parameters of the Time-Resolved Relaxation Experiments with SR Vesicles and Membrane Frag-ments

**preparation**	***τ*_1_**	***F*_1 _**	***τ*_2 _**	***F*_2 _**
caged H^+^				
vesicles	1.4±0.1 ms	0.030±0.001	31.9±0.1 ms	0.052±0.001
fragments	2.5±0.1 ms	0.025±0.001	34.2±0.6 ms	0.002±0.001
caged Ca^2+^				
vesicles	44.3±2.6 ms	0.021±0.001	302.6±6.7 ms	0.041±0.001
fragments	41.9±5.0 ms	0.005±0.001	272.6±8.0 ms	0.020±0.001
caged ATP				
vesicles	101.5±3.2 ms	0.083±0.001	5.4±0.03 s	0.200±0.001
fragments	105.0±2.9 ms	0.055±0.001	>6.0 s (n.s.)	0.004 (n.s.)

## References

[1] de Meis L (1985). Biochem Soc Symp.

[2] Yu X, Inesi G (1993). FEBS Lett.

[3] Yu X, Hao L, Inesi G (1994). J Biol Chem.

[4] Toyoshima C, Inesi G (2004). Annu Rev Biochem.

[5] Fibich A, Janko K, Apell H (2007). J Biophys J.

[6] Sørensen TL, Møller JV, Nissen P (2004). Science.

[7] Sørensen TL, Olesen C, Jensen AM, Møller JV, Nissen P (2006). J Biotechnol.

[8] Toyoshima C, Nakasako M, Nomura H, Ogawa H (2000). Nature.

[9] Toyoshima C, Nomura H (2002). Nature.

[10] Toyoshima C, Nomura H, Sugita Y (2003). Ann. N Y Acad Sci.

[11] Toyoshima C, Mizutani T (2004). Nature.

[12] Olesen C, Picard M, Winther AM, Gyrup C, Morth JP, Oxvig C, Moller JV, Nissen P (2007). Nature.

[13] Apell H-J, Marcus MM, Anner BM, Oetliker H, Läuger P (1985). J Membr Biol.

[14] Jørgensen PL (1974). Meth Enzymol.

[15] Peinelt C, Apell H-J (2005). Biophys J.

[16] Butscher C, Roudna M, Apell H-J (1999). J Membr Biol.

[17] Pedersen M, Roudna M, Beutner S, Birmes M, Reifers B, Martin H-D, Apell H-J (2002). J Membr Biol.

[18] Heilmann C, Brdiczka D, Nickel E, Pette D (1977). Eur J Biochem.

[19] Markwell MA, Haas SM, Bieber LL, Tolbert NE (1978). Anal Biochem.

[20] Schwartz AK, Nagano M, Nakao M, Lindenmayer GE, Allen JC (1971). Meth Pharmacol.

[21] Peinelt C, Apell H-J (2002). Biophys J.

[22] Peinelt C, Apell H-J (2004). Biophys J.

[23] Jørgensen PL (1974). Biochim Biophys Acta.

[24] Takayama M, Itho S, Nagasaki T, Tanimizu I (1977). Clin Chim Acta.

[25] Krainev AG, Ferrington DA, Williams TD, Squier TC, Bigelow DJ (1995). Biochim Biophys Acta.

[26] Brandt NR, Caswell AH, Brunschwig JP (1980). J Biol Chem.

[27] Deguchi N, Jørgensen PL, Maunsbach AB (1977). J Cell Biol.

[28] Bartolommei G, Buoninsegni FT, Moncelli MR (2004). Bioelectrochemistry.

[29] Tadini-Buoninsegni F, Bartolommei G, Moncelli MR, Guidelli R, Inesi G (2006). J Biol Chem.

